# Neuroplasticity: Insights from Patients Harboring Gliomas

**DOI:** 10.1155/2016/2365063

**Published:** 2016-07-05

**Authors:** Nathan W. Kong, William R. Gibb, Matthew C. Tate

**Affiliations:** ^1^Feinberg School of Medicine, Northwestern University, 303 E. Chicago Avenue, Ward Building 1-003, Chicago, IL 60611, USA; ^2^Department of Neurological Surgery, Feinberg School of Medicine, Northwestern University, 676 North Saint Clair Street, Suite 2210, Chicago, IL 60611, USA

## Abstract

Neuroplasticity is the ability of the brain to reorganize itself during normal development and in response to illness. Recent advances in neuroimaging and direct cortical stimulation in human subjects have given neuroscientists a window into the timing and functional anatomy of brain networks underlying this dynamic process. This review will discuss the current knowledge about the mechanisms underlying neuroplasticity, with a particular emphasis on reorganization following CNS pathology. First, traditional mechanisms of neuroplasticity, most relevant to learning and memory, will be addressed, followed by a review of adaptive mechanisms in response to pathology, particularly the recruitment of perilesional cortical regions and unmasking of latent connections. Next, we discuss the utility and limitations of various investigative techniques, such as direct electrocortical stimulation (DES), functional magnetic resonance imaging (fMRI), corticocortical evoked potential (CCEP), and diffusion tensor imaging (DTI). Finally, the clinical utility of these results will be highlighted as well as possible future studies aimed at better understanding of the plastic potential of the brain with the ultimate goal of improving quality of life for patients with neurologic injury.

## 1. Introduction

Traditionally, the brain has been considered a static organ, with little potential for plasticity [1]. This concept was centered on the notion that the brain is comprised of discrete sections, each controlling a specific function, and thus localized damage would result in largely irreversible and specific functional deficits. However, recent advances in neuroimaging and direct brain mapping have shown that the brain is capable of significant redistribution of function in response to injury [2–4]. It is believed that this remodeling, termed neuroplasticity, occurs continuously throughout life. Perhaps through similar mechanisms that are activated following brain injury, neuroplasticity is crucial for optimization of neuronal signaling [5].

Neuroplasticity has been extensively documented in developing children and ischemic stroke patients [6–14]. Glioma patients, a less frequently studied population, may represent another group that can give significant insight into neuroplasticity and its mechanisms. For example, it has been reported that lesions that occur in “eloquent” areas, such as Broca's or Wernicke's area, may not result in detectable language deficits [15–19], and in fact there have been several reports of resection of presumed critical speech and motor areas in glioma patients [20, 21]. It is believed that injury to these areas may be due to recruiting and reshaping neuronal connections, unmasking latent connections, or creating entirely new pathways [22–24]. On the other end of the plasticity spectrum, a modeling study in low-grade glioma patients suggested that when plasticity potential is exhausted, patients can exhibit seizure activity [25]. Additional research into how and where recruitment and reshaping are occurring in the brain may shed new light on principles governing plasticity of the adult brain. This review will give an overview of the mechanisms involved in neuroplasticity following brain injury, methods utilized to study plasticity, and fundamental questions that remain. Specifically, plasticity will be discussed in the context of patients harboring gliomas, as this group may present an optimal cohort to study neuroplasticity.

## 2. Mechanisms of Neuroplasticity

The study of neuroplasticity has traditionally focused on synaptic plasticity. Synaptic plasticity occurs in one or more synaptic junctions and is often mediated by the regulation of glutamate receptors—NMDA and AMPA glutamate receptors in particular [26, 27]. The modulation of NMDA and AMPA receptors due to differential neural stimulation is called long-term potentiation (LTP) [28]. LTP is the prevailing paradigm of microscopic neuroplasticity and is the primary mechanism underlying normal learning and memory [29]. This microscopic characterization of plasticity provides a physiological explanation for how synapses are continuously modulated during normal conditions.

In contrast, neuroplasticity following brain injury is less well understood, and although synaptic-level changes presumably play a role in redistribution of function, it is clear that larger-scale macroscopic plasticity plays an important role in cerebral recovery and reorganization following injury.

The brain displays a remarkable capacity for recovery after injury. While it has traditionally been assumed that the brain contains “eloquent” and “silent” areas, it is starting to become clear that the cerebral connectome, consisting of overlapping and independent networks, allows for a much more dynamic view of brain function and reorganization after injury. The understanding of canonical eloquent areas, such as Broca's area, has even been challenged to include larger and more connected networks [30]. This is to say, damage to an area traditionally considered critical for a given function may not cause irreversible damage, depending on the spatial and temporal features of the injury [31].

### 2.1. A Hierarchy of Plasticity

**Figure 1:**
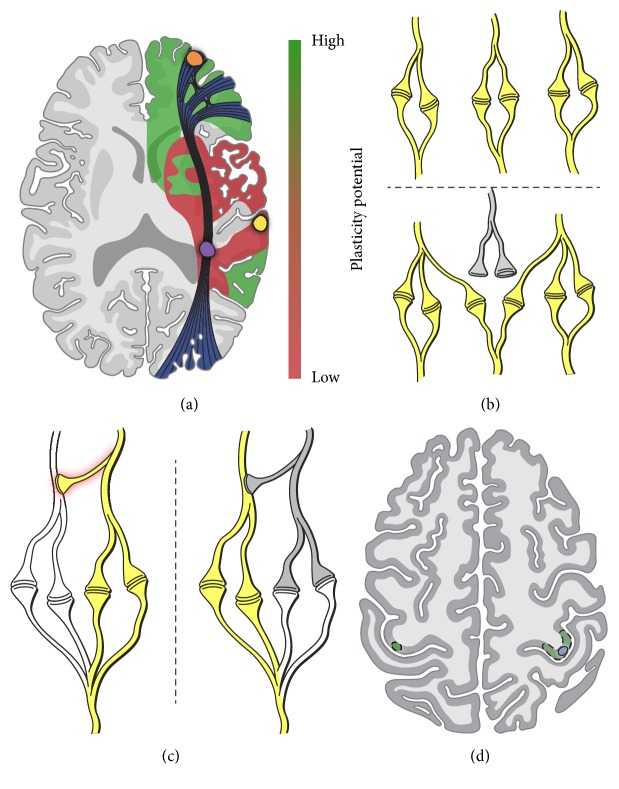
Mechanisms of neuroplasticity. Illustrated in panels (a)–(d) are the four primary mechanisms of neuroplasticity discussed in this review. (a) Plasticity hierarchy (adapted from Ius et al., 2011 [37]). Areas in green have a high potential for plasticity while areas in red have a low potential for plasticity. Three lesion examples are illustrated in this figure. A lesion in the anterior frontal cortex (orange) would likely exhibit a high plasticity potential due to its noncritical role in complex higher-order functions. Conversely, a lesion in the posterior superior temporal gyrus (yellow; Wernicke's area, a critical language network hub) or in the subcortical white matter tract (purple; inferior frontal occipital fasciculus, a major axonal pathway connecting receptive and expressive language areas) would be expected to have limited plasticity. (b) Cortical recruitment: when a cortical injury occurs (grey), perilesional synapses can be recruited to maintain synaptic integrity. (c) Cortical redundancy: redundant synapses are normally inhibited by interneurons (red shadow). Upon injury (grey), this inhibition is lost thus allowing for transmission of the redundant synapse. Instead of losing function, the redundant pathway compensates for the injured neurons. (d) Contralateral recruitment: a lesion (blue circle) in the hand motor area (green dashed region) can promote recruitment of the analogous contralateral hand area (green circle) to rescue hand function.

A discussion of the hierarchy of cerebral plasticity is important for the context of this review. The first distinction in this hierarchy is between cortical and subcortical plasticity. For the purpose of this review, the term subcortical refers to white matter axonal fiber below the cortical surface. While cortical injury has the potential to recover, lesions of the subcortical white matter tracts are likely irreversible [31, 32]. Clinically, regaining or reestablishing cortical representation of a given function can result in functional recovery. For example, in a Parkinson's disease patient undergoing chronic deep brain stimulation (DBS), neuroplasticity in sensory-motor and prefrontal/limbic regions is hypothesized to be the reason why the patient's tremor improved. In addition, ischemic stroke patients have been shown to change the organization of their motor cortex over a six-month span following ischemic injury resulting in improved motor function [7, 33]. Furthermore, glioma patients can remain functionally normal despite tumor infiltrating primary cortical regions. This plasticity is a result of cortical reorganization in response to the glioma as evidenced by direct electrocortical stimulation (DES) data [16, 31, 34, 35]. In contrast to these examples of cortical plasticity, the irreversible nature of subcortical tract damage results in a lack of functional recovery (Figure 1(a)), and there is even evidence that subcortical loss depresses the brain's ability to adapt to future insults [36]. The critical maintenance of the subcortical white matter pathways was demonstrated in a study that examined neuroplasticity capacity as a function of brain lesion location using DES data from 58 LGG patients. A more recent study by the same group looked at over 230 patients and found that subcortical white matter tracts were far less likely to display neuroplasticity if injured as compared to cortical regions, leading to the hypothesis that these core inviolate tracts compose the “minimal common brain” [31, 37].

The potential for cortical plasticity can be further classified according to the particular role of a given region. For example, a primary unimodal cortex, such as the primary motor area (M1) in the precentral gyrus, is essential for carrying out accurate motor commands, and thus injuries to M1 are likely to result in significant motor deficits with limited long-term recovery. Likewise, some higher-order cortical regions, such as Wernicke's area in the posterior superior temporal gyrus, assume such a critical role within the language network where plasticity is limited. Thus, injury to this area may result in permanent receptive aphasia. On the other end of the spectrum, areas such as the anterior frontal lobe that are involved in very widespread networks, such as those engaged in executive functions, are rather easily compensated for if damaged (Figure 1(a)), presumably due to multiple parallel pathways already engaged in that task (e.g., analogous contralateral frontal region). Between these extremes, areas such as the supplementary motor area (SMA) and Broca's area, which serve to integrate and “fine-tune” motor actions (e.g., bimanual coordination) and higher-order language processing (e.g., semantic and phonemic fluency), respectively, assume only an intermediate level of importance in these networks and therefore significant plasticity may be possible depending on the nature (size, kinetics, and location) of the brain injury. There is significant evidence for this hierarchy of cortical plasticity in humans, particularly data obtained from DES in low-grade glioma patients [37].

In summary, primary unimodal sites, critical network hubs, and major subcortical white matter bundles exhibit limited plasticity while cortical areas associated with complex, higher-order functions (i.e., broader networks) but not necessarily critical for that function have a high plasticity potential.

### 2.2. Cortical Recruitment and Redundancy

There are two main mechanisms proposed to explain cortical neuroplasticity. The first is the recruitment of adjacent neurons in the corresponding cortical layer (Figure 1(b)). As an example, synaptic plasticity has been shown to play a role in recruitment of adjacent cortical neurons following traumatic brain injury [38]. Furthermore, a cohort of spinal cord injury patients was found to have recruitment of adjacent cortical regions corresponding to the damaged spinal cord afferents [39].

Interestingly, cortical recruitment in neuroplasticity is not isolated to the areas immediately adjacent to a lesion. Recruitment of preexisting, contralateral connections may also occur. For example, a series of 12 stroke patients was studied by fMRI during rehabilitation, and the six patients who were most successful in regaining motor function displayed recruitment of hand motor cortex contralateral to the lesion (Figure 1(d)), while the six patients who were unable to recover hand function did not show recruitment of contralesional cortical sites [6]. Plasticity can also be actively induced in stroke patients to aid recovery. For example, a cohort of stroke patients implanted with an epidural electrode to stimulate the motor cortex in conjunction with their rehabilitation sessions had improved motor recovery, likely through the induction of Hebbian learning and plasticity [40, 41].

The other accepted mechanism in which cortical neuroplasticity occurs is through disinhibiting inhibitory interneuron connections [42]. This theory relies on the fact that there are redundant connections throughout the cortex and that there is significant elimination of functional synapses during development, known as synaptic pruning [43]. Upon injury, inhibited redundant circuits can be recruited to compensate for lost function (Figure 1(c)). As an example, signal redundancy was reportedly increased following partial blockage of primary auditory cortex function; in other words, an injury can promote the involvement of redundant, compensatory circuits [44]. Most directly, the unmasking of latent, redundant pathways in the human motor cortex has been shown via intraoperative direct electrocortical stimulation (DES) in glioma and arteriovenous malformation patients [45].

While both recruitment of adjacent synapses and latent redundant circuits may result in similar functional outputs, there are important conceptual differences. As discussed above, molecular signals from unpaired synapses guide the recruitment of adjacent neurites. This recruitment, while being acute, does take time depending on the surrounding neurite density and signal strength. Conversely, the unmasking of latent connections can occur immediately. The relative weighting of each of these mechanisms is consequently dependent on the type and severity of injury and may predict the degree and time course of neural recovery. This is consistent with findings in mice that show the difference in time course of dual-hemisphere lesions. Animals who were lesioned in both hemispheres coincidentally showed significant impairment in task performance, while rats who received the second hemispheric lesion 30 days after the first lesion showed no functional deficit [46]. These results suggest that contralateral compensation is a time-dependent phenomenon.

### 2.3. Neuroplasticity in Gliomas

An interesting model system for studying the clinical aspects of neuroplasticity is DES in the operating room, which allows the neurosurgeon to directly identify critical brain regions for a given function by virtually inhibiting a cortical or subcortical area. In patients who have undergone repeat craniotomy using DES for glioma resection, some areas identified as critical at the first surgery when reinterrogated at second surgery are no longer critical, thereby directly demonstrating cortical plasticity. Interestingly, in these repeat DES cases, larger tumor volumes showed a correlation with* less* functional reorganization [35], suggesting that larger gliomas may have already triggered compensatory neuroplasticity before the first surgery, so further functional reorganization would not be seen in the operating room. In addition, a higher rate of overt neurologic deficits is seen in high-grade glioma and acute stroke patients compared to slow growing low-grade gliomas. Thus, a more rapid injury to the brain may overwhelm the plasticity potential, while more chronic, indolent injuries could allow for maximal functional reorganization.

There is evidence for both ipsilateral and contralateral recruitment of healthy neural circuitry to compensate for glioma-induced injury. Ipsilateral recruitment appears to occur more acutely, most likely by the unmasking of latent connections. For example, short-term plasticity has been shown in the primary motor cortex of glioma patients during surgery [45]. On a longer-term scale, patients with resection of low-grade gliomas in the supplementary motor area (SMA) show the recruitment of the contralateral SMA to maintain function, and importantly the degree of this contralateral SMA recruitment was associated with faster recovery times [47], a finding which parallels the higher degree of functional recovery in ischemic stroke patients with contralateral hand knob involvement as discussed earlier (Figure 1(d)).

While cortical neuroplasticity is more conceptually straightforward in primary motor and sensory cortex, it has also been demonstrated in the eloquent language regions of the brain, such as Wernicke's and Broca's areas. A 2012 case report described a man who underwent two resections for a low-grade glioma in the left Wernicke area. The first operation identified the left Wernicke area as crucial for proper speech and thus the tumor was incompletely resected. Three and a half years after the first operation, a second operation identified Wernicke's area as no longer critical and thus the tumor could be fully removed [48].

Patients who have had two surgeries with DES present the best cases to study how cortical and subcortical neuroplasticity changes in response to pathology, surgery, chemotherapy, and radiation over time. A recent study presented findings from a series of 18 glioma patients who had repeat DES-based cortical mapping as part of routine surgical care, an average of four years apart. In six patients, motor and language cortical regions that had previously elicited responses were no longer active during the second surgery. However, there were no corresponding motor and speech deficits on neurological examination, indicating that the motor and language functions were being controlled by different cortical regions during the second surgery [35]. Another report by Duffau and colleagues described three patients who each underwent serial glioma resections involving DES. The first important finding from this study is that although there was tumor infiltration of presumed sensory, motor, and language cortices, no patients exhibited overt neurologic deficits. Thus, there must be compensatory plasticity mechanisms that unfold during tumor growth. On the other hand, it was noted that functional areas, detected by DES, could be found within the tumor parenchyma. In these cases, bulk tumor was left behind in order to preserve neurologic function. This finding of intratumoral functional areas was more prevalent in the initial operations. At the second operation, 1-2 years later, all three patients displayed reorganization of cortical sites, one each in motor, somatosensory, and language areas, as detected by a lack of functional consequence in the second operation that was present in the first operation. Specifically, cortical function either within the tumor or at the obvious tumor margins in the first operation was no longer functional at the second operation. This finding is significant because it allows for more complete tumor resections at a second operation while minimizing patient morbidity throughout the treatment period. It is also suggestive that surgery itself can induce neuroplasticity and that this plasticity can benefit the patient by redirecting function away from the growing tumor site [34]. From a neuroscience perspective, the lack of functional deficit seen in patients with LGGs suggests a key paradigm in cortical neuroplasticity. Since LGGs grow relatively slowly, the brain has time to recruit significant compensatory mechanisms to maintain function. Thus, the slower time course of this pathology is an excellent lens through which we can study neuroplasticity and glean insight into its mechanisms and governing principles.

### 2.4. Factors Affecting Plasticity

Plasticity is believed to be both a developmental and a compensatory mechanism of the body. As with other common mechanisms, there are a variety of factors that enhance or diminish the reshaping of the brain. Age is a major factor that affects the ability of the brain to respond to injury. Animal models have indicated that crucial proteins for cortical plasticity, such as microtubule-associated proteins, are significantly decreased in the hippocampus and cerebellum as the animal ages [49, 50]. The kinetics of the lesion are another factor that plays a role in neuroplasticity. Patients with slow growing lesions (i.e., low-grade gliomas) tend to have fewer deficits than those with fast growing lesions (i.e., acute stroke). For example, a computational model developed by Keidel et al. found that there was a difference in the pattern of reorganization between strokes and low-grade gliomas [51]. As mentioned above, location of a brain lesion is another variable in the brain's response. In addition to cortical versus subcortical regions, it has been shown that higher level cognitive functions (i.e., visuospatial attention) tend to have greater reorganization potential than primary functions such as movement [52]. Recently, clinicians have attempted to take advantage of the plastic potential of the brain in order to help patients recover function after an injury [53, 54]. Early rehabilitation can induce more plasticity compared to no intervention or delayed interventions [53]. Lastly, neuroplasticity can be affected by sex and genetics, which may in part explain an individual's differing responses to similar injuries [55, 56].

## 3. Techniques for Studying Neuroplasticity

Here, we discuss the major modalities that can be used to study neuroplasticity. The advantages and disadvantages of each technique will be examined as well as how it can be applied to the field.

### 3.1. Direct Electrocortical Stimulation (DES)

**Figure 2:**
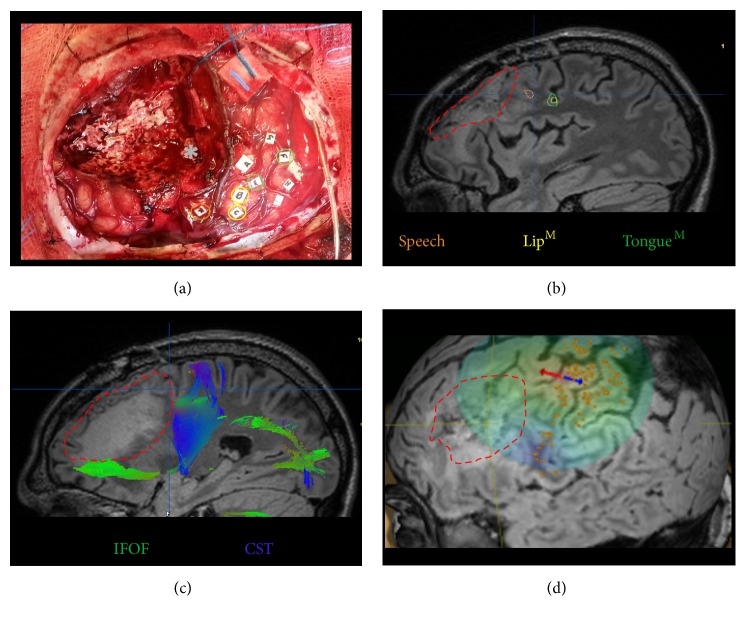
Neuroplasticity mapping methods. Illustrated in panels (a)–(d) are various brain mapping techniques performed on a 30-year-old right-handed male with low-grade glioma intraoperatively (a) and prior to surgery ((b)–(d)). The approximate tumor border is shown (red dash) in panels (b)–(d). (a) Postresection intraoperative photograph of functional sites elicited by direct electrical stimulation: tags B/C lip movement (yellow); tags E/F tongue movement (green); tag K speech difficulty (orange). Note the close correlation between cortical language (orange), lip motor (yellow), and tongue motor (green) sites obtained by fMRI (panel (b)). Other tags shown denote face motor sites. At the inferior/posterior border of the resection cavity, stimulation of the IFOF (gray asterisk) caused speech disturbance. (b) Functional MRI (fMRI) demonstrating language activation (green) at the junction of pars opercularis and precentral gyrus and lip (yellow) and tongue (green) motor activation in the inferior precentral gyrus. (c) Diffusion tensor imaging (DTI) demonstrating two major white matter bundles in close proximity to the tumor: the inferior frontal occipital fasciculus (IFOF, green) that transmits semantic language information and the corticospinal tract (CST, blue) which conveys descending primary motor information. (d) MRI-navigated transcranial magnetic stimulation (TMS) of the hand motor area of the precentral gyrus elicited overt muscle contraction.

DES is performed intraoperatively by neurosurgeons in order to identify critical functional areas. The methods for DES have been discussed in other papers [30, 57]. Briefly, local anesthesia is administered followed by a craniotomy under awake conditions. Once the cortical surface is exposed, the surgeon stimulates the brain surface using a bipolar probe (stimulation parameters: 60 Hz, biphasic, 1 msec pulse duration, and 2-3 sec stimulus duration). For motor and sensory mapping, positive findings are noted, that is, movement or dysesthesias of the hand, respectively, during stimulation. For language mapping, the stimulation is used to create a temporary and reversible “virtual lesion” during counting or object naming paradigms in order to predict the functional outcome if that particular area was to be resected [58]. Thus, if a language deficit is elicited during stimulation, the surgical team denotes this area as “eloquent” for language and will not resect the area even if it involves a tumor. Using this method, surgeons can pinpoint the areas that can and cannot be removed with a spatial resolution of approximately 1 cm, resulting in the maximal removal of affected brain tissue while minimizing permanent neurologic decline (Figure 2(a)).

In addition to allowing for maximal resection and thus improved clinical outcomes, it also provides a precise way to map the critical functional areas of the brain, that is, the epicenters of functional brain networks. Thus, intraoperative DES remains the gold standard technique for establishing a critical role for a given brain region.

Currently, DES is being used to map areas of the brain that absolutely cannot be resected, termed the “minimal common brain.” This brain atlas can be helpful in surgical decision-making for patients that cannot tolerate awake craniotomies [37]. DES has also challenged the traditional tenets of brain organization by showing that language areas may not be localized strictly to Broca's and Wernicke's area, even if the tumor itself is distant from those regions [30]. DES can also be performed multiple times on the same patient, giving a unique insight into how the patient's brain remodels after resection [35].

While DES has many advantages, its major disadvantage is the invasiveness of the procedure. With the exception of primary motor cortex mapping, DES patients must be awake during the procedure, thus rendering some patients ineligible [30, 57]. In addition, DES requires that patients have lesions that necessitate surgery and by definition is not performed in normal healthy patients. Also, only areas that are exposed can be mapped using DES; thus, distant ipsilateral and/or contralateral functional contributions cannot be investigated. Finally, the precise changes elicited by DES, particularly in the case of interruption of function, may not be limited to the area directly under the stimulus [59]. Thus, DES should be considered as one of several tools capable of studying neuroplasticity. It will be necessary in the future to improve the fidelity of DES while also incorporating data from other techniques.

### 3.2. Functional Magnetic Resonance Imaging (fMRI)

In the last 25 years, fMRI has gained popularity and is the most utilized noninvasive method for mapping function in the brain [60]. fMRI takes advantage of the principle of increased neural activity leading to an increase in local concentration of deoxyhemoglobin that is utilized in blood-oxygen-level dependent (BOLD) imaging [60]. By measuring the concentration of deoxyhemoglobin, neural activity during tasks, such as finger tapping or picture visualization, can be measured and visualized on coregistered anatomic MRI (Figure 2(b)).

Given that fMRI provides another way to map functional areas of the brain, a large number of studies, the majority involving normal patients, have investigated brain regions involved in movement, language, cognition, empathy, and even rest [61–64]. In studies examining neuroplasticity in patients harboring lesions, fMRI can provide a window into how the brain adapts to injury. For example, fMRI data can be obtained pre- and postoperatively in glioma patients after surgical resection in order to study changes in functional anatomy [47]. To date, the results of the fMRI studies have been inconsistent; some reports demonstrate significant changes in motor and language plasticity while others claim no changes are found [5, 18, 65–67]. While the correlation of fMRI with DES is not always consistent, perhaps due to fMRI indicating all regions involved in a given function as opposed to only the critical regions, fMRI does provide an important noninvasive technique to assist in decision-making for surgery or to study the activation patterns of the brain in patients who cannot be examined via DES. For example, in patients with low-grade gliomas near primary motor regions, serial fMRI may be used to demonstrate the time point at which peritumoral function has reorganized to more distant areas and thus the surgeon may elect to return for a second resection at that time. In addition, fMRI allows simultaneous study of all regions of the brain and thus does not have the spatial constraint of DES.

The major drawback of fMRI is that it is not a direct measurement of neural function or activity. By using deoxyhemoglobin as a proxy for neuronal activity, the specificity and sensitivity of the test lead some to conclude that fMRI cannot yet replace DES as a reliable way of functional mapping in the brain [68]. fMRI data is also inherently noisy due to variability from heart rate and patient movement [69]. This leads to a low-resolution image where it is difficult to determine whether changes in neuronal activity have indeed occurred or whether the results are simply a product of slight shifts in perfusion. Finally, standard fMRI has limitations in temporal resolution, as one can only study features at a time scale equal to or slower than blood flow changes, which are typically slower than electrical (i.e., primary) changes.

fMRI techniques have also been applied to patients at rest, without any engagement in activities. Termed resting-state fMRI (rsfMRI), this technique has been remarkably consistent among individuals, suggesting that rsfMRI may allow resolution of basic network nodes and edges [70]. rsfMRI is now being used to study plasticity both in healthy patients and in those with lesions [71–73]. For example, a recent study in chronic stroke patients, who had undergone a 4-week rehabilitation session, was able to restore connections between ipsi- and contralateral motor areas as measured by rsfMRI [72]. This showed that rsfMRI could be used to measure the effective connectivity (which should be distinguished from anatomic connectivity; see below) between nodes and how they change in response to injury and therapy.

### 3.3. Connectivity Measures

More recently, significant work has been put into understanding the white matter connections between areas of the brain and the contribution to functional networks [74]. In addition to resting-state fMRI connectivity metrics discussed in the previous section, there are two additional imaging techniques frequently used to investigate white matter (i.e., structural) connections: corticocortical evoked potential (CCEP) and diffusion tensor imaging (DTI).

CCEP is a method where one area of cortex is stimulated electrically (in a manner similar to DES) and subsequent potentials are recorded in another brain region, thus establishing the notion that an electrical connection exists between the areas, whether it is monosynaptic or polysynaptic [75]. Based on the details of the recorded waveform, it can be established that the two cortical regions are indeed specifically connected (versus passive electrical transmission through the brain). CCEP has similar drawbacks to DES in that it is an invasive test and requires surgery. In addition, demonstration of electrical connectivity by CCEP does not definitively establish the notion that the stimulated circuit is utilized physiologically.

DTI takes advantage of water displacement along white fiber tracts, where it was noted that water diffuses faster along the direction parallel to the tracts than perpendicular to them [76]. This subtle change could be visualized on MRI, thus providing a noninvasive way of looking at subcortical connections (Figure 2(c)). Recently, a group of researchers used DTI to measure the changes that resulted from transcranial direct current stimulation therapy of stroke patients [77]. DTI showed increasing descending motor tract anisotropy following therapy, indicating that motor improvement following the therapy had structural underpinning [77]. The researchers hypothesized that the increased fractional anisotropy may indicate increased motor fiber alignment, myelination, and/or overall fiber integrity. Future work hopes to combine DTI with other techniques to better define the structural correlates of neuroplasticity.

### 3.4. Transcranial Magnetic Stimulation (TMS)

TMS is a technique where electrical current can be applied noninvasively to the cortical surface to provoke positive effect, such as hand muscle activation with stimulation of primary motor cortex, or to create a temporary lesion in the cortex, for example, speech arrest with stimulation of ventral premotor cortex (Figure 2(d)) [78]. Thus, TMS can be used to map functional areas in a manner similar to DES. Advantages of TMS include the ability to map functional areas in both hemispheres of the same patient and to investigate normal healthy controls. In addition, TMS offers a platform to noninvasively study brain plasticity in an individual patient over time and has given insights into how the brain adapts to acute and chronic lesions [19, 79–81]. Despite the obvious theoretical advantages of TMS, the major disadvantage is reliability; with the exception of motor mapping, the optimal parameters for virtual lesioning are not well established, with a relatively high number of false positives when compared to DES mapping of similar functions such as language [82]. In addition, TMS of more lateral areas such as the temporal lobe can cause patient discomfort with contraction of the temporalis muscle. Nonetheless, TMS remains an exciting tool for studying and modulating plasticity.

### 3.5. Electroencephalography (EEG) and Magnetoencephalography (MEG)

EEG and MEG are two methods that directly measure cortical electrical activity noninvasively. Studies have shown that EEG can robustly measure cortical connectivity in both healthy and lesion models [83, 84]. One study showed that decreased connectivity as measured by EEG correlated with decreased motor skill in stroke patients [83]. In another report, researchers found that the imitation based therapeutic intervention for aphasic patients (IMITATE) led to increased slow-wave activity measured by EEG, showing the acute changes that occurred following the therapy [85]. EEG can also be combined with TMS to study connectivity in a manner similar to CCEP [86, 87]. While these methods hold promise as a component of multimodal approaches to studying brain plasticity, each has significant limitations. The main drawback of EEG is the signal-to-noise ratio due to recording already small amplitude signals from the brain surface that are additionally attenuated by the skull and scalp.

By measuring magnetic fields induced by cortical electrical activity, MEG has shown changes in cortical activity following injury and rehabilitation, especially in spinal cord injury patients [39, 88]. In one study, researchers used MEG to show that motor imagery training in tetraplegics resulted in decreased cortical activity variability (as compared to their pretest activity patterns) that mimicked their healthy counterparts [39]. MEG is primarily limited by its cost and relative lack of data compared to other methods discussed.

### 3.6. Electrocorticography (ECoG)

ECoG utilizes the same principles as EEG except that electrodes are applied directly on the cortex following a craniotomy, which allows for greater sensitivity and precision compared to scalp EEG. By examining activity from implanted ECoG electrodes in the context of brain-machine interface (BMI), it has been demonstrated that the BMI learning resulted in initial increases in prefrontal, premotor, and posterior parietal cortical activity with subsequent decrease in activity once the subjects became proficient [89]. These results highlight the power of ECoG to precisely map the spatial and temporal profile of brain plasticity. Limitations of ECoG include the invasive nature of direct brain surface recording, which introduces time constraints and limits spatial access. In addition, as with fMRI, MEG, or EEG, ECoG does not necessarily allow differentiation of critical from involved brain regions. However, ECoG can be combined with DES to establish the distinguishing ECoG “signature” of DES-defined network nodes. This multimodal approach holds promise for understanding basic organizing principles of normal and plasticity-induced brain networks.

### 3.7. Positron Emission Tomography (PET)

PET can also be applied to studying neuroplasticity. By utilizing the direct relationship of increased activity with increased (typically) glucose metabolism, PET imaging can provide insights into functional variation that may occur after a patient has sustained a lesion. Using PET imaging, one study found that when left sided glioma patients performed a verb-generation test, they had more left frontolateral activation of areas that were not classically language centers, as well as increased activity of the right frontolateral hemisphere when compared to healthy controls [90]. Using PET imaging that is specific for GABA receptors, one study showed neuroplastic changes that occurred in GABAergic receptor availability following a subcortical stroke [91]. PET imaging is limited by its poor temporal resolution (even more so than fMRI). Measuring cerebral blood flow takes 90 sec and then cannot be repeated for at least 6 min [92]. In addition, PET is mildly invasive because radioactive substances must be inhaled or injected for the study [92].

### 3.8. Neuropsychological Assessment

Neuropsychological testing can be used to determine the extent of “initial” neurological deficit following a brain insult and subsequently document the degree of functional recovery as well as the time scale of such changes as a function of patient and lesion parameters. Also, these tests can be helpful in delineating truly redistributed function from compensatory behaviors. Thus, in conjunction with the other methods discussed in this review, neuropsychological analysis can serve as an objective outcome measure of plasticity in an attempt to determine which variables (lesion volume, acuity of lesion, anatomic location of lesion, etc.) explain why certain patients can adapt to lesions while others suffer loss of function [93].

## 4. Clinical Significance

While being able to detect changes in the brain can provide insight into how the brain adapts, the ultimate goal of biomedical research is to improve patient outcomes. By analyzing neuroplasticity in patients, healthcare providers can now provide more specific and aggressive therapies to even the most daunting of cases. Below, we describe some of the potential ways in which neuroplasticity could be harnessed to improve patient care, using the settings of ischemic stroke and glioma surgery as examples.

Following stroke, clinicians have attempted to harness the plasticity potential of the brain to improve motor, language, and cognitive deficits. For example, patients undergo a variety of physical rehabilitation interventions such as body-supported treadmill, bilateral arm training, or using robotic devices. Patients often have improved outcomes after these sessions relative to control conditions, inferring that plasticity mechanisms have been accelerated during the training [53]. In a large meta-analysis, the Cochrane Stroke Group concluded that physical rehabilitation of any kind had a beneficial effect on recovering function and mobility [94]. Repetitive transcranial magnetic stimulation (rTMS) is another therapy that has been employed based on principles gleaned from neuroplasticity studies. Stroke not only affects the local infarct region but also can secondarily disrupt the inhibitory function of the directly damaged region, leading to cortical excitability. Thus, rTMS may have dual roles for stroke patients: high-frequency (positive) stimulation to the affected region can improve excitability and low-frequency (negative) stimulation on the contralateral side may reduce excitability [95]. A large meta-analysis showed positive effects of rTMS on finger motor ability after subcortical stroke [96]. The analysis included studies with high-frequency stimulation in the affected hemisphere and low-frequency stimulation in the unaffected hemisphere. Both methods resulted in motor activity improvement [96]. Taken together, these data argue that an intelligent combination of focused rehabilitation in conjunction with cortical stimulation based on sound understanding of fundamental principles of neuroplasticity may present an effective strategy for accelerating and improving functional deficits from stroke or other neurologic disorders.

In the context of glioma surgery, by combining imaging modalities (fMRI, DTI, and TMS) with direct cortical and subcortical stimulation, surgeons are now able to resect larger areas of affected brain tissue than once thought possible (e.g., Broca's area, hand motor region, and Wernicke's area), while minimizing neurologic deficits [97]. This apparent paradox of being able to resect assumed “eloquent” areas can be possible (if validated by DES intraoperatively) due to (a) inadequate understanding of “normal” functional networks (e.g., recent data demonstrating that the brain region serving as the final common output for speech is not Broca's area but rather ventral premotor cortex within the inferior precentral gyrus [30]); (b) functional compensation (e.g., patient unaware of homonymous hemianopsia); and (c) mechanisms of lesion- or (prior) surgery-induced neuroplasticity such that the area is no longer functionally required [17]. Often, however, the entire lesion cannot be removed during a single surgery (i.e., DES demonstrates critical functional areas at the tumor site), but the recognition of the ability for the brain to change has led to a multistage surgical approach where a second surgery is performed after brain reorganization has occurred (perhaps documented over time with serial fMRI scans or TMS sessions). During the second surgery, after confirming plasticity with DES intraoperatively (i.e., a cortical area required at the first surgery is no longer critical), a greater extent of resection can be achieved without compromising neurologic function [98, 99].

## 5. Future Concerns

Researchers and clinicians alike are interested in learning how the brain adapts to change, both in the normal and in the pathologic states. An understanding of the governing principles of neuroplasticity could have important translational impact on patients by improving quality of life and survival. However, there are still a number of fundamental questions that remain. What are the structural and functional changes underlying recovery? Are there consistent patterns of plasticity? How can we trace the progression of reorganization? Could we perhaps actively modulate plasticity in a novel and specific way to accelerate normal plasticity mechanisms? Are all forms of plasticity beneficial? [58, 68, 78]. At the heart of these unresolved issues is the need to rigorously characterize the particular parameters that promote or dissuade neuroplasticity and how each factor impacts behavior. For example, there has been some suggestion that the kinetics, location, and type of brain injury can determine the extent and type of plasticity that occurs [15]. Along this line, future work could compare plasticity in slow growing lesions, such as low-grade gliomas or benign tumors, with acute lesions (stroke, malignant glioma, and traumatic brain injury). Combining these findings with the effect to injury location and size will move us toward the goal of translating basic plasticity mechanisms into clinical benefit. Finally, although the vast majority of previous reports detailed in this review have dealt with plasticity of language and sensorimotor function, the ultimate goal is to expand our knowledge of functional plasticity into higher-order systems (attention, memory, and social cognition), which will lead to novel treatments and ultimately a greater quality of life for patients and their caregivers.
